# Rethinking grit in Chinese higher education: toward a purpose-based account of perseverance

**DOI:** 10.3389/fpsyg.2026.1791633

**Published:** 2026-04-13

**Authors:** Tao Zhong

**Affiliations:** Henan Normal University, Xinxiang, China

**Keywords:** China, cultural context, higher education psychology, purposeful grit, university student development

## 1 Why traditional grit may fall short among Chinese university students

Since Duckworth's initial formulation, grit, defined as perseverance of effort and consistency of interests for long-term goals, is one of the most influential constructs in educational psychology (Duckworth et al., 2007; Duckworth and Quinn, 2009). Grit has been widely adopted in research, policy discourse, and educational practice as a psychological explanation for academic performance, educational attainment, and resilience (Hodge et al., 2018; Zhao and Wang, 2023; Derakhshan and Fathi, 2024). Within higher education, grit is often implicitly treated as a universally adaptive trait: The more students demonstrate sustained, undiverted effort, the stronger their anticipated academic and developmental outcomes.

However, this assumption becomes increasingly problematic when applied uncritically to the Chinese university context. Chinese students are frequently characterized by exceptionally high levels of sustained academic effort and endurance in the face of pressure (Li, 2017; Xu et al., 2023). At the same time, mounting evidence documents elevated levels of academic burnout, emotional exhaustion, anxiety, and hopelessness among Chinese university students (Lew et al., 2019; Zhang et al., 2022; Liu et al., 2023). This paradox, where sustained effort accompanies compromised wellbeing, contradicts the assumption that grit is uniformly adaptive, indicating that traditional grit may not guarantee wellbeing (Quinn et al., 2024).

I argue that this paradox reflects a fundamental limitation in how grit has been conceptualized and applied. Specifically, grit captures the strength and durability of effort but remains largely silent about the meaning, direction, and motivational quality of that effort. Moreover, consistency of interests within grit does not guarantee that long-term goals are experienced as meaningful or self-endorsed. Rather, it focuses on persevering through distraction and may reflect stable engagement or interest without necessarily involving reflective purpose or value integration. This conceptual limitation becomes critically important when grit is applied to specific cultural and institutional settings. In contexts where long-term goals are self-chosen, flexible, and aligned with personal values, this omission may be less consequential. In China's highly structured, competitive, and socially embedded educational system (Yu et al., 2018), however, the absence of a purpose-sensitive lens risks misclassifying maladaptive endurance as psychological resilience.

## 2 Defining purposeful grit: toward meaning-regulated perseverance

Purposeful grit is proposed as a conceptual refinement of grit that foregrounds the motivational meaning underlying sustained effort, rather than treating perseverance as inherently adaptive. While traditional grit emphasizes how persistently individuals pursue long-term goals (Duckworth et al., 2007; Wang et al., 2021), purposeful grit addresses a prior and more fundamental question: what gives sustained effort its psychological direction and adaptive value. At its core, purposeful grit refers to the capacity to sustain long-term effort because one's goals are experienced as meaningful, self-endorsed, and worth continued investment, even in the face of difficulty. This definition emphasizes that perseverance is not merely a quantitative property (how long or how intensely effort is sustained), but a qualitative motivational process shaped by purpose.

Two individuals may display similar levels of effort and sustained engagement, yet differ substantially in purposeful grit depending on whether their sustained effort is guided by internally endorsed purpose or externally imposed obligation. In this sense, purposeful grit redefines what counts as psychologically adaptive perseverance. Effort becomes adaptive not simply because it is sustained, but because it is anchored in goals that individuals experience as meaningful and identity-consistent.

In the following subsections, I elaborate the conceptual foundations of purposeful grit by situating it within self-determination theory and Confucian moral psychology, clarifying its distinct analytic focus within Chinese higher education. Specifically, the reconceptualization draws on these two complementary theoretical traditions to clarify how purpose is internalized, sustained, and translated into long-term effort within the sociocultural ecology of Chinese higher education.

### 2.1 Self-determination theory: purpose as the internalization of values

Self-determination theory posits that motivation varies in quality depending on the degree to which goals are autonomously endorsed and integrated into the self (Deci and Ryan, 2002; van Schie et al., 2019; Ryan et al., 2024). When individuals internalize the value of a goal, effort becomes more sustainable and psychologically adaptive. From this perspective, effort is maintained not through coercion or fear of failure, but through a sense of personal ownership and coherence between action and identity.

Purposeful grit aligns closely with this internalization. It reflects a form of perseverance that emerges when long-term goals are not only pursued consistently, but also experienced as meaningful expressions of one's values and sense of self. In educational contexts characterized by intense evaluation and social comparison, such as Chinese universities, students often engage in prolonged effort under conditions of controlled motivation. While this may produce short-term performance gains, it frequently undermines wellbeing and leads to motivational exhaustion over time (Yu et al., 2018). Purposeful grit, by contrast, represents autonomous perseverance. It captures how purpose functions as a motivational anchor that stabilizes effort across challenges, setbacks, and periods of low external reward.

Conceptually, purposeful grit is not to replace existing motivational constructs, but to clarify a specific gap at their intersection. While intrinsic motivation and self-determination theory emphasize the degree to which goals are autonomously endorsed (Ryan et al., 2024), and the construct of purpose in life addresses the presence of meaningful long-term aims (Kim et al., 2022; Giang et al., 2024), these perspectives do not directly account for how such meaning is translated into sustained effort over time. Purposeful grit is proposed to bridge this gap by conceptualizing perseverance as a process regulated by internalized meaning, highlighting not only whether goals are valued, but whether and how such values sustain adaptive effort. In this sense, purposeful grit shifts the analytic focus from the presence of motivation to the regulation of sustained effort by meaning over time.

### 2.2 Confucian moral psychology: purpose as ethical and collective orientation

While SDT provides a powerful account of motivational internalization, it does not fully capture the moral and collective dimensions of purpose that are central to Chinese cultural traditions. Confucian moral psychology conceptualizes human development as a lifelong process of self-cultivation (xiushen), in which personal effort gains meaning through its contribution to family, community, and society (qijia zhiguo pingtianxia). Within this framework, perseverance is not merely instrumental but ethically oriented in that effort is valued for its alignment with moral ideals and social responsibility. Perseverance, in this view, is inherently relational as to persist is to honor familial sacrifices, respect one's teachers, and prepare oneself to serve the collective good (Wang and Liu, 2010; Yeh and Chou, 2024; Taylor and Zhang, 2025). This helps explain why Chinese students often frame academic struggles in collective terms, interpreting hard work as a duty rather than merely a personal choice.

In Chinese higher education, this cultural logic remains salient. Students are often encouraged to persist not only for personal success, but also to fulfill filial expectations, contribute to collective prosperity, and uphold social harmony (Zhou, 2014; Han and Cheung, 2025). These values can provide a powerful sense of direction, transforming effort into a morally meaningful endeavor. However, when such expectations are imposed without internalization, they may also generate pressure, guilt, and emotional strain.

Purposeful grit bridges this tension by emphasizing internalized moral purpose rather than externally imposed obligation. It acknowledges that perseverance in the Chinese context is frequently embedded within relational and collective goals, while insisting that such goals must be psychologically integrated to support resilience rather than distress. In this sense, purposeful grit resonates with Confucian ideals of self-cultivation while remaining attentive to individual agency and wellbeing.

## 3 Implications for education and mental health, and future research directions

### 3.1 Rethinking academic perseverance in educational practice

In many Chinese universities, continued effort is implicitly equated with diligence, discipline, and moral worth. Students who continue working despite exhaustion are often praised as resilient, while disengagement or reconsideration of goals may be interpreted as weakness. From the perspective of purposeful grit, this evaluative framework is incomplete and potentially harmful.

Educational practice should therefore shift from reinforcing how much students endure to examining why they persist. Purposeful grit encourages educators to treat perseverance as psychologically adaptive only when students can articulate a meaningful rationale for their sustained effort. This reframing legitimizes reflective disengagement from misaligned goals and reduces the moralization of overexertion. In classroom settings, instructors can support this shift by encouraging students to reflect on the personal and social significance of their academic pursuits, rather than focusing exclusively on performance indicators.

### 3.2 Designing pedagogical environments that cultivate purposeful grit

Purposeful grit is unlikely to emerge in learning environments that emphasize competition, ranking, and extrinsic rewards alone. Instead, it is fostered through pedagogical practices that help students connect sustained effort with meaning and contribution. Autonomy-supportive teaching, relevance-oriented curriculum design, and opportunities for socially meaningful engagement are particularly important in this regard.

Instructors can frame difficult coursework not merely as a test of endurance, but as preparation for addressing real-world problems or contributing to societal needs. For example, a statistics course for psychology students could use datasets from actual community mental health surveys, asking students to analyze patterns that could inform local intervention strategies. Service-learning, community-based projects, and research experiences that connect academic knowledge to social impact can transform continued effort from an obligation into a purposeful commitment. These practices do not reduce academic rigor; rather, they redefine rigor as sustained effort in the service of meaningful goals, which is key to purposeful grit.

### 3.3 Purpose-oriented interventions as mental health promotion

Interventions aimed at strengthening students' purposeful grit may have downstream benefits for mental health by transforming how effort is experienced. Narrative-based approaches, such as life story reflection and guided visualization of one's “future self,” can facilitate this shift. For example, students might be asked to visualize the person they hope to become and the contributions they aspire to make, then write a letter from that future self, reflecting on how current academic efforts have shaped their life trajectory. Such exercises help students reinterpret present struggles as part of a meaningful developmental journey rather than as evidence of inadequacy.

Importantly, these interventions should not prescribe purpose or impose moral ideals. Instead, they should support students in articulating personally and culturally meaningful reasons for continued effort. In the Chinese context, this may involve helping students integrate personal aspirations with relational and societal values in ways that feel self-endorsed rather than coercive. When students experience their perseverance as purpose-driven, effort becomes a source of coherence and resilience rather than depletion.

### 3.4 Future directions for empirical research

Several avenues for future empirical investigation emerge from this conceptualization of purposeful grit. The construct may be understood as multidimensional, involving at least two interrelated components. The first is sustained effort over time, reflecting the behavioral persistence traditionally emphasized in grit research. The second is purpose regulation, referring to the extent to which sustained effort is guided by goals that are experienced as meaningful, self-endorsed, and identity-consistent. To assess these dimensions empirically, rigorous scale development and validation research is needed.

Beyond scale development, researchers should consider interaction-based indices that capture the alignment between sustained effort and purpose regulation. For example, purposeful grit may be evaluated as the degree to which high levels of perseverance co-occur with high levels of purpose regulation, rather than merely as components in isolation. Such alignment is expected to predict psychological wellbeing more strongly and to distinguish purpose-driven perseverance from effort sustained without internalized meaning.
